# Assessing the Generalizability of Foundation Models for the Recognition of Motor Examinations in Parkinson’s Disease

**DOI:** 10.3390/s25175523

**Published:** 2025-09-04

**Authors:** Christopher Gundler, Alexander Johannes Wiederhold, Monika Pötter-Nerger

**Affiliations:** 1 Institute for Applied Medical Informatics, University Medical Center Hamburg-Eppendorf, Martinistr. 52, 20246 Hamburg, Germany; 2 Department of Neurology, University Medical Center Hamburg-Eppendorf, Martinistr. 52, 20246 Hamburg, Germany

**Keywords:** Parkinson’s disease, movement data, self-supervised learning, foundation model, generalizability, human activity recognition

## Abstract

Current machine learning approaches focusing on motor symptoms in Parkinson’s disease are commonly trained on small datasets and often lack generalizability from developmental setups to clinical applications. Foundation models using large, unlabeled datasets of healthy participants through self-supervised learning appear attractive for such setups with limited samples, despite the potential impact of motoric symptoms. Acting as an exemplar, this study aims to evaluate the robustness of fine-tuned models in recognizing movements related to motor examinations across datasets and recording setups. Accelerometer data of 51 participants with Parkinson’s disease in three different training and fine-tuning setups were used to tailor the general model to the disease. Training the model on pre-trained weights, both partially (F_1_ = 0.70) and fully (F_1_ = 0.69), statistically significantly outperformed training the model from scratch (F_1_ = 0.55) in a nested cross-validation. For evaluation, the model’s ability to process data recorded from 24 patients in clinic was tested. The models achieved lower mean F_1_ scores of 0.33 (train from scratch), 0.43 for full, and 0.48 for partial fine-tuning, but demonstrated improved generalizability and robustness regarding the orientation of sensors compared to training from scratch. Utilizing foundation models for accelerometer data trained on healthy participants and fine-tuned for clinical applications in movement disorders appears as an effective strategy for optimized generalizability with small datasets.

## 1. Introduction

Parkinson’s disease (PD) is the most rapidly growing neurological disease with a doubling of its prevalence from 1990 to 2016 [1]. This progressive, neurodegenerative disorder is characterized by specific motor symptoms such as bradykinesia, muscle rigidity, tremor, and postural instability as well as various non-motor symptoms [2]. The disease progresses with a consecutive change in symptom load, urging a need for thorough clinical reassessment and constant therapy adjustments for effective management [3]. Consequently, for adequate adjustment of therapy, long-term monitoring is advisable. In clinical routine, the physician usually assesses the patient in the outpatient clinic every 3–6 months and receives only a single moment impression, but no objective measures over the last weeks. Using movement data from smartphones, wearables, and related devices for motor assessments in PD could fill that information gap. Additionally, increasing research activities focus on on-demand, closed-loop stimulation systems to improve PD symptoms such as deep brain stimulation or biochemical sensing for pharmacological analytics [4,5] on a moment-to-moment basis. There, external wearables appear attractive as a biomarker of the motor state, e.g., to detect freezing of gait episodes [6]. Accordingly, device-aided movement tracking has become widespread in research and discussed with its challenges and opportunities [7].

Optimized for activity recognition, sensor data from accelerometers, gyroscopes, and magnetometers are commonly used in related studies [8]. Some studies focus on experimental setups in which patients perform specific tasks designed to elicit symptoms, and these tasks are then assessed automatically [9,10]. Other authors try to derive disease-relevant indicators for monitoring sensor assessments in real-world settings, capturing a more naturalistic representation of daily activities but without rater-based matching [11,12]. In both cases, the continuous sensor signal must often be segmented into different parts of activity, depending on the research question of interest. For example, detecting freezing of gait only makes sense when a patient is moving, and assessing some items of the Movement Disorder Society Unified Parkinson’s Disease Rating Scale (MDS-UPDRS) only makes sense when the related movement is performed. These segmentations themselves may have different origins; physicians, scientists, or even patients often annotate them manually during studies. In real-world data, extracting those segments automatically for healthy participants is possible and is still improved in current research under the name of human activity recognition. In case of research regarding PD, related work is more limited; for example, Yue et al. [13] describe a similar setup. Some authors utilize their own data, while others use existing models trained on public data and assume they will still perform for patients despite their characteristic motoric symptoms [14].

### The Issue of Generalization into Clinical Reality

The limited methodical considerations regarding the precise method for obtaining the associated activities appear amplified given the requirements regarding the robustness and generalizability of the approaches. This challenge is multifaceted. Firstly, the overall data volume is constrained. Although efforts have been made to consolidate diverse data sources for larger cohorts, the available data remains comparatively limited, particularly when compared to tasks such as general activity recognition. The problem becomes even worse as the available samples differ significantly between cohorts. Often, observational studies with multitudes of measurements recorded in home setups are based upon self-reported outcomes [15]. Mixing these unsupervised real-world data with samples recorded and annotated by experienced clinicians will likely increase the included variance significantly. Lastly, the missing standardization of sensors, their placements on the body, temporal resolutions, preprocessing methodologies applied to raw data, and similar technical factors represent a considerable challenge. Designing data-driven algorithms capable of accommodating these multifaceted challenges and establishing them in clinical reality represents a substantial hurdle [16].

Challenges regarding the amount or quality of labeled data are not limited to the clinical domain and require methods that can transfer knowledge from larger, sometimes healthier, populations and datasets. Transfer learning and, more recently, self-supervised learning [17] allow models to learn generic representations from vast pools of unlabeled data, which can then be re-used for specialized tasks with limited labeled data—an approach that has shown promise in the biomedical domain [17,18,19]. In the context of Parkinson’s research with its limited amount of data, some related work has assessed the methodology for numerous sensory modalities suitable for the symptoms. Based on videos, the technology has been used to obtain general gait patterns to assess the gait of patients with PD [18]. For the same objective, the technology has shown benefits for electroencephalography data [19], voice data [20], brain scans [21], or given extracted skeletons [22]. Focused on movement data, the technology proved its use for assessing disease prognosis [23], for assessing specific motor examinations [24], or for detecting anomalies of walking and freezing of gait [23,25].

Recently, Yuan et al. [26] demonstrated that self-supervised foundation models pre-trained on accelerometer data could be fine-tuned to achieve significant improvement in activity recognition with a limited number of labels. Unlike similar work (i.e., [27]), they assessed the generalizability of the knowledge not only for healthy patients. While not the primarily focus of their work, they reported up to 135% performance gain when they fine-tuned the model instead of training it from scratch on a single dataset with accelerometer data of PD patients. However, the authors did not address generalizability across multiple PD datasets with varying recording conditions, sensor placements, and labeling procedures. Such cross-dataset robustness is critical for real-world clinical deployment, wherein models must cope with substantial between-dataset heterogeneity. Based upon their seminal findings, the aim was to empirically test two interconnected research hypotheses as follows:

1. Fine-tuned foundation models trained through self-supervised learning on accelerometer data of healthy participants enhance the recognition of activities associated with motor examinations conducted by PD patients across datasets and recording paradigms;
2. The fine-tuned models show increased robustness to varying recording conditions and different data preprocessing commonly observed between studies.

Through the corresponding findings, the study contributes insights into the potential usage of “general” representations of accelerometer data for use in PD research and increase robustness regarding the usage of similar systems in clinical reality.

## 2. Materials and Methods

### 2.1. Data

#### 2.1.1. Datasets

To evaluate the potential of pre-existing knowledge regarding accelerometer data derived from healthy participants for the recognition of motor examinations in PD patients, three datasets were employed. Two publicly available datasets, namely the Clinician Input Study (CIS-PD) [28] and the Levodopa Response Trial [29], were utilized as instances of observational studies conducted mostly in an ambulant setting [28,30,31,32]. The third study includes data of motor disturbances from PD patients in the Parkinson’s Clinical Movement Assessment (PACMAN) study, which was conducted by the Neurology Department at the University Medical Center Hamburg-Eppendorf [33].

Data from 24 participants of the CIS-PD study were included with their clinical assessments and recorded ambulatory long-term measurements with hospital visits (4 study sites in the United States), mainly at the beginning and the end of the trial. While not being at a study site, participants performed self-assessed ratings via a mobile phone application. Patients wore an Apple Watch Series 2 and only tri-axial accelerometer data were recorded and transferred to a mobile phone application, where a low-pass filter and summation of absolute acceleration in 5 and 30 s window intervals were performed [28].

The Levodopa Response Trial [29] was conducted on four consecutive days with selected items of the third part of the UPDRS on the first and the last day of the study. Participants repeated the tasks 6 to 8 times. The participants wore at least three devices: their smartphone, GeneActiv and Pebble. All of these devices were worn throughout the entire study period, while recording daily activities during the second and third days at the participants’ homes. The annotated accelerometer data of the GeneActiv device (ActivInsights Ltd., Kimbolton, United Kingdom [34]), worn on the most affected hand of the patients (upper limb), were included in the study. The data of these 27 participants were collected on two study sites in the United States. Further information regarding the devices and study setup could be found in the original publication [30].

As an example of data recorded in an entire clinical setup, sensor recordings of the PACMAN study originally collected for assessing methods of motor disturbances at the University Medical Center Hamburg-Eppendorf, Department of Neurology, Germany, were utilized [33]. In a configuration striving for similarities to clinical routine, a physician handed out an Apple Watch Series 6 to hospitalized patients with PD and conducted up to three assessments of seven selected items (3.3 rigidity, 3.4 finger tapping, 3.5 hand movements (tight fist), 3.6 rotation of hands, 3.9 arising from chair, 3.10 walking (gait), 3.17 rest tremor amplitude) of the UPDRS’s third part per day. Those procedures were repeated while the therapy of the patients was adjusted for a maximum of two weeks. A total of 24 of these participants available during conduction of this study were included [33]. All procedures performed in the PACMAN study were in accordance with the ethical standards of the institutional research committee and with the 1964 Helsinki Declaration and its later amendments or comparable ethical standards. The collection of measurements from the patients was approved by the Ethics Commission of the Ärztekammer Hamburg with the ID 2022-100846-BO-ff. Informed consent was obtained from all individual participants included in the PACMAN study.

The study encompassed 24, 27, and 24 participants of the CIS-PD, Levodopa Response Trial, and the PACMAN study, respectively. Table 1 provides an overview of the participants and available samples for each activity class, revealing a notable variation in the number of labels across datasets. Particularly, the ambulant observational studies (CIS-PD and Levodopa Response) amassed up to 17,000 samples for specific labels through self-assessments. In contrast, the clinic-based study at UKE, involving assessments by a physician, yielded considerably fewer samples.

#### 2.1.2. Data Preprocessing

The datasets represent a collection of the typical paradigms that could be found in research regarding motor symptoms of PD. To ensure interoperability at least regarding the format and labels of the accelerometer data, we utilized a unified data structure to map all datasets to a common database structure [35]. None of the datasets were specifically designed for recognizing activities belonging to motor examinations. To obtain measurements for the different classes of activity, the raw signal was segmented into areas according to the given activity class labels. We split these areas containing only one kind of movement into segments of 10 s with a maximum overlap of 50%. This choice was guided by compatibility with the previous work and to reduce information loss at segment boundaries. No further preprocessing was conducted to maintain a representative sample of the measurements recorded in varying recording setups.

In collaboration with an experienced neurologist, we selected four different classes of activity that are useful for assessing motor symptoms of PD. Besides classical human activities like standing, sitting, and walking, we included the rotation of hands, as it is part 3.6 of the UPDRS. All the individual classes of labels between the different datasets were mapped to these “dataset-independent” classes. Whenever a dataset included a mixture of tasks, like moving from sitting to standing, we excluded those. The remaining activities that were of limited interest or unavailable within the other datasets were mapped to the class “other” and contained mostly a mixture of daily activities like drinking or writing. The set of those classes represented the foundation for the subsequent analysis.

### 2.2. Model

#### 2.2.1. Foundation Model

As a recent example of a foundation model for accelerometer data trained through self-supervised learning, the publicly available model by Yuan et al., based on roughly 700,000 days of movement data from more than 100,000 healthy participants, was chosen for comparison [26]. This model employs a ResNet-V2 architecture with 18 layers and one-dimensional convolutions, which processes temporal windows of 10 s at a sampling rate of 30 samples per second. The model is designed to accept tri-axis accelerometer data as input, without the necessity for supplementary contextual information. To ensure direct comparability of results, our study replicated the experimental paradigm reported by the original authors. After the “embedding layer” with 1024 neurons, a dense layer with 512 neurons and the final output layer with five neurons, each corresponding to a target class, was added. The Softmax activation function was then applied to obtain class probabilities.

#### 2.2.2. Fine-Tuning the Foundation Model

Depending on the three setups for evaluation, the foundation model underwent training or fine-tuning within a nested cross-validation framework, utilizing combined data from the CIS-PD and the Levodopa Response Trial. This process involved five outer test folds paired with eight inner validation folds, allocating 70% of the dataset for training, 10% for validation, and 20% for testing. The stratification of folds ensured that no participant’s data was included in multiple folds, thus preserving the integrity of the evaluation. The training sessions explored three distinct learning rates (0.01, 0.001, and 0.0001), covering a range of reasonable defaults with a fixed batch size of 1024 determined by the used GPU resources, prototypical for clinical workstations. An early stopping criterion was employed to halt training if no improvement was observed in the validation set over 50 epochs. To reduce the complexity of hyperparameter analysis, advanced learning rate adaptation strategies were not implemented. The Adam optimizer facilitated the optimization process, utilizing cross-entropy loss to reduce the discrepancy between predicted logits and five target classes.

For evaluation, the multiclass F_1_ score, defined as the average harmonic mean of precision and recall across all classes was used. This metric was chosen because it is compatible with the work by Yuan et al. [26]. This metric ranges from 0 to 1, with the latter indicating optimal classifier performance. The F_1_ score on the validation set served as the basis for early stopping and as the criterion for selecting the best model weights during training. During the testing phase, the weights from that point were used to calculate the F_1_ score on the test set.

The training was conducted using a single consumer-grade NVIDIA A100 graphics processing unit, highlighting the model’s potential for straightforward replication and application in clinical settings, even with limited computational resources.

### 2.3. Evaluation

**Figure 1:**
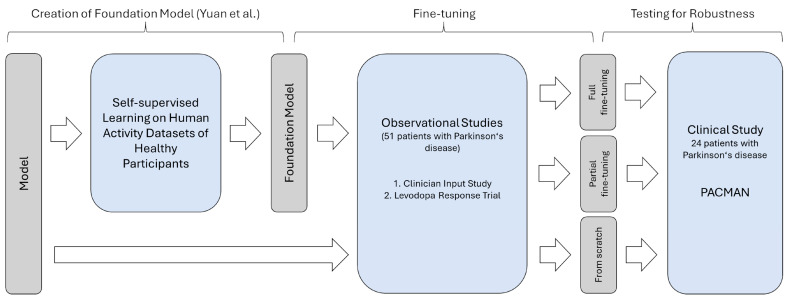
Overview of the evaluation procedure to test for the impact of different fine-tuning paradigms. PACMAN, Parkinson’s Clinical Movement Assessment.

The evaluation was conducted in two consecutive steps guided by the research hypotheses. For the first question, the training of an algorithm was simulated as it would be conducted in a scientific routine. Given the CIS-PD and the Levodopa Response Trial, the nested cross-validation was used both to obtain a suitable model for the task and an estimate of the performance on unseen data as a measure of generalizability through the incorporation of the test sets. For the sake of assessing the influence of learned representations, the training was run in three paradigms (Figure 1):

- Training from scratch: The first evaluation was based on training the network from scratch. In this setup, the pre-trained weights of the self-supervised model were not used at all. Instead, a random initialization of the network according to the utilized PyTorch library (version 1.13) took place. Accordingly, the deep-learning architecture is trained as it would be if other data besides the data in the training set were unavailable. This condition represents the baseline and could be used to study effects such as the appropriateness of the model structure. However, the risk of overfitting is high.
- Partial fine-tuning: The second training paradigm, partial fine-tuning, was based on training only the last layers of the network for predicting the presence of motor examination. The remaining layers with their pre-trained weights serve as feature extractors and were frozen. While the number of parameters requiring training is the fewest and the risk of overfitting is reduced accordingly, the other layers may not account for a changed distribution in the input data, given the non-healthy study population.
- Full fine-tuning: The third evaluation, the full fine-tuning, consisted of training the full network while using the existing weights of the foundation model as a starting point. While the model may fully adapt to the changed input data, previously extracted representations of movements could be reused. However, overfitting might affect the performance on unseen data, given the size of the network and the few training samples.

For all the paradigms, we assessed the potential positive impact of utilizing the learned representations for the chosen task of recognizing the motor examinations.

The second hypothesis was tested regarding increased robustness to varying recording conditions by testing the model against the clinical data (PACMAN). As one would expect given the heterogeneous datasets, the dataset did not contain measurements for the label “standing”. Subsequently, the obtained performance was compared to those estimates derived through the test folds in the previous analysis. Instead of relying on additional metrics besides the F_1_ score, the resulting confusion matrices were analyzed directly.

Besides this testing for generalizability between the recording environment, the impact of the recording setup, like the orientation of the sensor or scale of the data, was investigated. This was inspired by typical incompatibilities previously identified [35]. For that, the clinical PACMAN dataset was deliberately modified to generate two additional modified datasets. Through a simple linear transformation, the reported recordings according to the standardized SI unit and the common “g unit” (scaled dataset) were simulated. Additionally, the measurements from different cohorts were aligned, given the axis explaining most variance during the walking tasks, calculated through a principal component analysis [36] (rotated dataset) for measuring the impact of the orientation of the device.

### 2.4. Statistical Analysis

A classical *t*-test, with Welch’s modification to account for potential changes in variances, was employed to test for significant performance differences between different models. Results are presented as mean ± standard deviation, and a threshold of statistical significance was set at *p*  <  0.001 to ensure robustness of the findings.

## 3. Results

### 3.1. Impact of Training Procedures for Fine-Tuning

The trained models, when evaluated on the test folds during the nested cross-validation, exhibited average F_1_ scores of 0.55 ± 0.06 when trained from scratch, 0.70 ± 0.02 when partially fine-tuned, and 0.69 ± 0.09 when fully fine-tuned, respectively (mean ± standard deviation). Across all experiments, models trained from scratch converged in an average of 144 ± 66 epochs, while partially and fully fine-tuned models required 128 ± 47 and 137 ± 62 epochs, respectively. Supporting the first hypothesis, training the model on pre-trained weights, both partially and fully, statistically significantly outperformed training the model from scratch.

### 3.2. Influence of Learning Rate for Fine-Tuning

**Figure 2:**
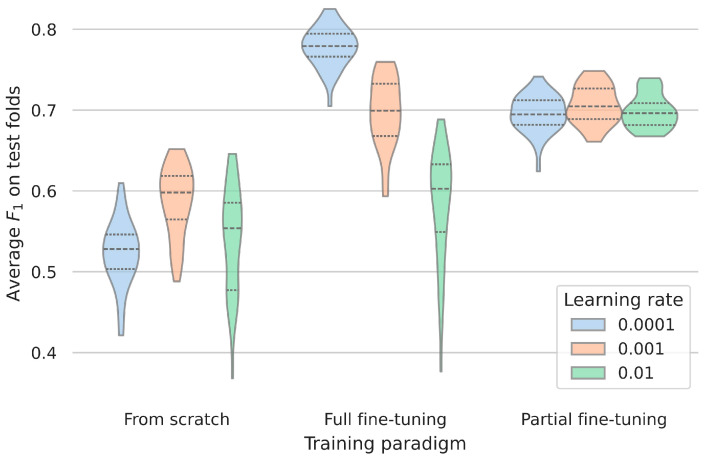
The impact of the learning rate on the two observational studies combined across the different training paradigms. When trained from scratch, the model failed to achieve a reasonable performance on the validation set. For the fine-tuning paradigms, the influence of the learning rate was higher (in full fine-tuning) or lower (in partial fine-tuning). F_1_ = 1 corresponds to the optimal performance. The dotted lines indicate the quantiles of the data.

The impact of learning rates during evaluation differs significantly (Table 2). When comparing learning rates of 0.0001, 0.001, and 0.01, training from scratch resulted in average F_1_ scores from 0.52 to 0.59. The peak performances on the test folds were obtained during full fine-tuning with scores above 0.58. In this case, the smallest learning rate was significantly better suited than the other cases. In partial fine-tuning, the effect of the learning rate was effectively mitigated and the performances were highly comparable (Figure 2). The frozen weights in the latter setup appear to let most of the models reliably converge to slightly sub-optimal values.

### 3.3. Assessing the Robustness and Generalizability of the Clinical Dataset

**Figure 3:**
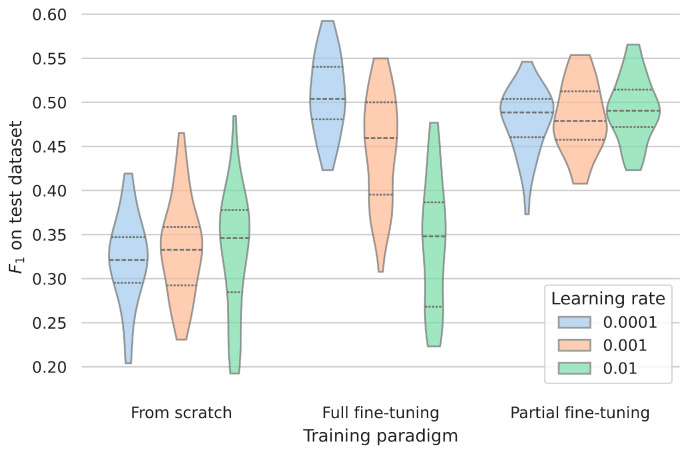
The performance of the models trained on cohort studies when applied to PACMAN as the test dataset for different learning rates and training paradigms. Despite the learning rate, the fine-tuned models show significantly better results than the models trained from scratch. F_1_ = 1 corresponds to the optimal performance. The dotted lines indicate the quantiles of the data.

In the literature regarding motor symptoms, the obtained test score within (nested) cross-fold validation is commonly treated as an indicator of the proposed method’s generalizability. To assess the effects of representations encoded within the foundation model for generalization in clinical setups more realistically, the models were applied to the PACMAN dataset. There was a considerable drop in absolute performance compared to the test folds’ performances. The trained models achieved mean F_1_ scores of 0.33 ± 0.06 when they were trained from scratch on the observational data, 0.43 ±  0.09 after being fully fine-tuned, and 0.48 ± 0.04 after partial fine-tuning (Figure 3).

In terms of relative performance difference, the fine-tuned models demonstrated considerably better generalization capabilities compared to the model trained de novo. Building upon representations learned from healthy participants resulted in statistically significantly better performance, commonly between 15 and 25%. Depending on the chosen fine-tuning paradigm, the learning rate had varying influence; a learning rate too large, when all weights could be modified, appeared to overwrite the previously learned representations completely, leading to similar performance as models only trained on the original data. When overfitting was not possible due to freezing most of the weights, the learning rate did not make a significant difference.

**Figure 4:**
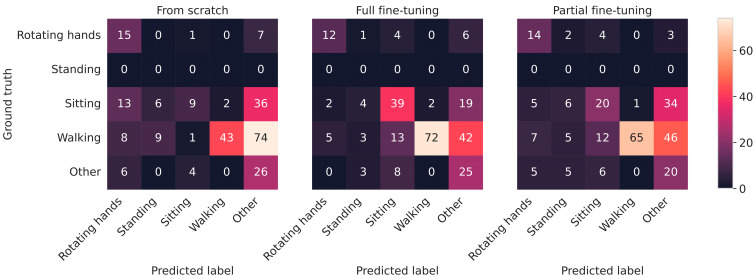
Confusion matrices of the best-performing models on the test folds from the three different training setups. The classes of the ground truth specify the correct class, while those below correspond to the prediction. Accordingly, an optimal classifier would only have values on the diagonal.

For a more comprehensive understanding than afforded by singular evaluation metrics, Figure 4 provides a visual depiction of the confusion matrices on the clinical dataset. For each training paradigm, the model with the highest score on the test folds of the observational dataset was chosen and applied to the data from the clinic.

While the sample size of the data recorded in a clinical context might be small compared to other similar datasets, some differences are observable. When considering the three distinct training paradigms, movements inherently present in the foundation model, such as walking, were consistently well recognized in the fine-tuned models. For movements not explicitly familiar to the model, such as hand rotation, the selected models exhibited no discernible advantages. While the dataset did not contain any samples of standing to mirror the differences in available labels across related datasets, this did not contribute to a large fraction of the wrongly classified activities.

### 3.4. Evaluating the Robustness According to Recording Setups

**Figure 5:**
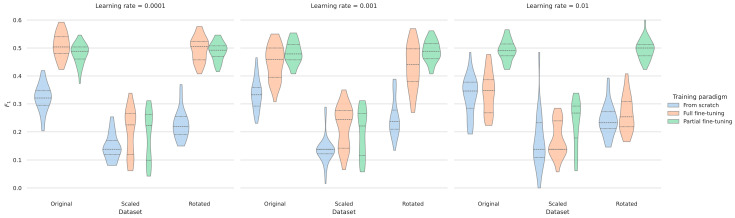
The performance of the models trained on the cohort studies when applied to the PACMAN as the test set for different learning rates and training paradigms. While all models show a considerable drop in accelerometer data of a different unit, the fine-tuned models show significantly better results when the sensor is artificially rotated. F_1_ = 1 corresponds to the optimal performance. The dotted lines indicate the quantiles of the data.

To assess the models’ robustness regarding technical changes in recording setups beyond the nature of the study, the test sets were modified as described in the Section 2 to simulate measurement data in different units and rotated sensors. The results are provided in Figure 5.

All training paradigms exhibited significantly lower performance compared to the original data when confronted with the linearly scaled dataset, a simulation mirroring the discrepancy between the “g unit” and the SI unit for accelerometer data. However, the fine-tuned models showed similar (or sometimes even slightly better) performance when the data was rotated to simulate a sensor with a different orientation. The only exception to this was the fully fine-tuned model optimized with a high learning rate, which apparently overfitted the data and lost its advantages of the learned representations in comparison to a model trained from scratch.

## 4. Discussion

The limited amount of data and the disparate recording setups between studies focusing on motor symptoms of PD pose significant challenges for the sustainable development of the area of research. Enhancing the generalizability of proposed approaches is crucial for transitioning solutions from laboratories to clinical settings. The goal of this study was not to differentiate movements of PD patients from those of healthy controls, but rather to recognize specific motor examination activities in PD patients, benefitting from robust, generalizable movement representations learned on large healthy cohorts across different datasets.

### 4.1. Benefits of Utilizing Data from Healthy Participants for Movement Disorder Research

In line with the previous research by Yuan et al. [26], fine-tuning foundation models previously trained on accelerometer data originally collected from healthy participants significantly improved the classification of movements associated with tasks performed by PD patients. Despite the inherent differences in movement patterns between both populations, employing pre-trained weights within the deep learning framework resulted in performance improvements on test folds of up to 25% compared to training models solely on disease-specific datasets. However, this improvement over multiple training and validation datasets is significantly lower than the reported performance in the case of a single dataset. This finding highlights the danger of overfitting to the specific recording conditions and is evident in the choice of the hyperparameters, too. While the best performance was observed in the entirely fine-tuned model, the training process and its associated hyperparameters, like the learning rate, must be tightly observed to not “overwrite” the previous knowledge and overfit. Forcing the model only to adopt the parts responsible for classification efficiently reduced the danger of overfitting; however, it did not result in the best possible performance. The finding that the choice of learning rate critically affected performance, especially in full fine-tuning, likely reflects the interplay between step size and convergence landscape. Larger learning rates may have caused the optimization to miss global minima or become trapped in local minima, particularly given the limited data and early stopping strategy.

Given the computational efficiency of this approach, the additional complexity of the training setup does not pose a significant barrier for adoption in clinics. Even considering the resource-intensive nature of the meta-analyses in this study due to nested cross-validation, the process of fine-tuning a single network is manageable with a consumer-grade GPU, making it accessible and feasible for widespread use for related problems in hospitals.

### 4.2. Evidence for Increased Robustness Regarding Recording Setups

This study’s findings suggest that the increased robustness not only holds during cross-validation but also when tested on a dataset recorded within the clinic. The observed degradation in performance was even larger than anticipated given the results on the test folds, prompting caution when interpreting results from nested cross-validation as accurate estimates of generalizability. Despite this, the generalized representations provided by fine-tuned models yielded significantly better results than models trained solely on disease-specific data. Specifically, the fine-tuned models demonstrated a 15–20% improvement over the scratch-trained models. The findings suggest that the performance “scales” between different setups. The relative differences between training paradigms and learning rates remained relatively consistent, indicating that optimization on the validation set could translate effectively to better results even across different datasets. This property is highly advantageous, suggesting that effective cross-validation can guide better model tuning even across varying data conditions.

The model’s robustness to artificially induced sensor rotations is encouraging, suggesting that representations learned by the foundation model may encode invariant features across axis permutations and device placements, a desirable property for real-world wearable deployments. By contrast, performance degradation during testing unit scaling (simulating changes from “g unit” to SI unit) likely indicates sensitivity to absolute input magnitude distribution. Accordingly, improved interoperability remains necessary. When models are trained from scratch, they do not exhibit this robust behavior, further providing evidence for fine-tuned models’ capability to generalize under varied recording setups. However, it must be noted that orientation robustness was assessed using mathematically simulated data, which may not fully replicate the complexity of human-worn sensor placements in uncontrolled environments. Future studies should consider explicitly collecting datasets with known, systematically varied sensor orientations.

### 4.3. Utility of the Proposed Model for Recognizing Motor Examinations

To reduce the number of possible confounders for the analysis, a rather simple approach towards recognizing motor examinations was chosen. Despite the challenges of classifying specific movements such as hand rotation, which were not familiar to the model, the classification of movements like walking and sitting—prevalent in the data by the healthy participants used for training the model—was more successful despite the associated motor symptoms. This supports the use of large-scale pre-training for developing more reliable clinical monitoring tools. The absolute performance obtained through fully fine-tuning the model and testing it on the clinical data is certainly a valid starting point, but further improvements must be considered before applying such a model in the clinical context. Naturally, the obtained labels extracted from small windows should be aggregated given a meaningful temporal context, i.e., through the usage of attention mechanism. The selection of suitable targets remains important given the apparent challenges for complex movements resulting in the higher misclassification rates for the class “other”, as reflected in the off-diagonal elements of Figure 4′s confusion matrices. Overall, the utilization of data from healthy participants led to significantly better results and should likely be utilized in similar challenges.

### 4.4. Limitations and Future Work

Given the obtained insights within this study, we are certain that re-using the representation of accelerometer data will be more often used for tasks related to motor symptoms of PD. However, in the context of generalizability being the special focus of this work, some limitations should be considered in future work.

Firstly, the investigation focused on a single foundation model for accelerometer data chosen for its technical soundness, state-of-the-art performance, and computational efficiency. Future research could explore a variety of foundation models to further improve generalizability and performance. The large search space for optimizing hyperparameters, such as learning rates, batch sizes, and optimizers, poses a challenge. Optimization of the model architecture should be considered, too. While only two layers were trained in the partial fine-tuning setup to minimize overfitting, adding more layers may yield further improvements. Advanced transfer learning strategies, such as gradual unfreezing, adapter layers, or per-layer learning rates, should also be considered. Although this study addressed learning rates specifically due to their significant impact, future studies with greater computational resources might explore a broader range of hyperparameter configurations. Existing literature may already provide the first hints for such an optimized implementation [37].

Furthermore, this study concentrated on accelerometer data, a representative yet not exclusive modality for analyzing movement disorders. Incorporating additional sensor modalities, such as gyroscopes or magnetometers, in a multimodal framework, may further enhance performance. Additionally, considering temporal segments beyond fixed lengths could offer better real-world applicability, potentially combining multiple predictions into a unified score through additional post-processing steps.

## 5. Conclusions

In summary, the use of foundation models for accelerometer data holds the potential to significantly improve the performance on movement-related tasks in PD across recording setups. In the specific example of recognition of motor examinations under study, the additional knowledge embedded through self-supervised learning always led to significantly better results despite the presence of motor symptoms. The technology significantly improved the robustness of the approach despite discrepancies between observational and clinical datasets and recording variations. While it cannot entirely resolve all generalizability issues, it provides a meaningful step towards more robust, clinically applicable models. Continued research and development, focusing on broader datasets and diverse model architectures, will be essential for bridging the remaining gaps in research regarding motor symptoms and bringing these advancements into clinical routine.

## Figures and Tables

**Table 1:** Patient characteristics and absolute number of annotated tasks available for every type of movement across the different cohorts. The first two studies contain far more measurements while the third dataset from the clinical domain contains far fewer variables. On the clinical set recorded at UKE, no tasks were selected while the patient was standing.

	Levodopa Response Trial	Clinician Input Study	PACMAN Study
Participants	27	24	24
Age range	50–84 years	36–75 years	49–79 years
Average age (SD)	67 (±9) years	63 (±10) years	65 (±8) years
Rotating hands (UPDRS 3.6)	4912	908	23
Other movements	15,172	3012	36
Sitting	2077	436	66
Standing	2077	418	0
Walking (UPDRS 3.10)	7010	950	135

UPDRS, Movement Disorder Society Unified Parkinson’s Disease Rating Scale; PACMAN, Parkinson’s Clinical Movement Assessment.

**Table 2:** F_1_ scores for the three learning rates on the two observational studies combined across the different training paradigms.

Learning Rate	F_1_ Score (±SD)		
	From scratch	Full fine-tuning	Partial fine-tuning
0.0001	0.52 ± 0.04	0.78 ± 0.02	0.70 ± 0.02
0.001	0.59 ± 0.04	0.70 ± 0.04	0.71 ± 0.02
0.01	0.54 ± 0.06	0.58 ± 0.07	0.70 ± 0.02

F_1_ = 1 corresponds to the optimal performance.

## Data Availability

All the model weights trained during the analysis and the code required for replicating the study on own data are publicly available on GitHub (https://github.com/UKEIAM/de.uke.iam.parkinson.activity [accessed on 31 August 2025]) and the scientific data repository of the University of Hamburg (http://doi.org/10.25592/uhhfdm.13995 [accessed on 31 August 2025]).

## Abbreviations

The following abbreviations are used in this manuscript:

CIS-PD	Clinician Input Study
PACMAN	Parkinson’s Clinical Movement Assessment
PD	Parkinson’s disease
MDS-UPDRS	Movement Disorder Society unified Parkinson’s Disease Rating Scale
